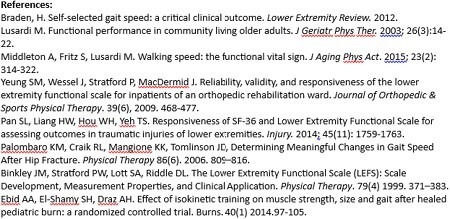# 75 Qualitative Improvement Project Assessing Changes in Perceived Physical Function and Gait Speed in Burn Patients

**DOI:** 10.1093/jbcr/irae036.067

**Published:** 2024-04-17

**Authors:** Lynn Fossen, Allison Davis, Taylor Trapp

**Affiliations:** Loyola Medical Center, Chicago, Illinois; Loyola University Medical Center, Maywood, IL; Loyola Medical Center, Chicago, Illinois; Loyola University Medical Center, Maywood, IL; Loyola Medical Center, Chicago, Illinois; Loyola University Medical Center, Maywood, IL

## Abstract

**Introduction:**

The impact of lower extremity burns can extend far beyond the initial injury. Currently there is minimal research regarding outcome measures to track patient recovery in acute care. The purpose of this project is to utilize standardized outcome measures to delve into critical aspects of recovery, assessing changes in perceived physical function and gait speed for patients with lower extremity burns within a hospital stay. By exploring these parameters, we aim to gain a comprehensive understanding of how individuals with lower extremity burns adapt and recover, shedding light on potential interventions and strategies that can improve their quality of life and mobility.

**Methods:**

Physical therapists completed the 5-meter walk test and Lower Extremity Functional Scale with patients who had lower extremity burns within 48 hours of their initial physical therapy evaluation and within 48 hours of their hospital discharge. Patients included in the data collection are those with lower extremity burns over the age of 18 who are hospitalized for more than 48 hours. Exclusion criteria included patients who are non-ambulatory, those with cognitive impairments, patients hospitalized due to suicide attempt, patients who undergo amputations, and patients with preexisting neurological diagnoses affecting their independence. Data was collected and analyzed using Microsoft Excel. Normative data including means and standard deviations were reports.

**Results:**

Data was collected from Dec 2018 to March 2020 and July 2022 to present. There are 85 operative patients, 31 female and 54 male and 28 nonoperative patients, 10 female and 18 male. The average age for operative patients is 48.5 years and nonoperative patients is 52.8 years. The average TBSA of the operative group is 9.8 and nonoperative is 4.8.The average gait speed at evaluation for the operative group was 0.62 m/s and 0.65 m/s at discharge. The average LEFS at evaluation for the operative group is 32.6 and 36.9 at discharge. The average gait speed at evaluation for the nonoperative group is 0.37 m/s and 0.67 m/s at discharge. The average LEFS at evaluation is 27.7 for the nonoperative group and 39 at discharge.

**Conclusions:**

The 5-meter walk test and LEFS are simple and informative outcome measures to perform in acute care burn rehabilitation. These measures give insight into quality of life and gait capabilities following lower extremity burns.

**Applicability of Research to Practice:**

Understanding the changes in perceived physical function and gait speed following lower extremity burns is crucial in allowing us to assess the immediate and long-term impact of these injuries on an individual’s mobility and well-being. This QI project can inform healthcare professionals about the specific challenges patients face during recovery leading to tailored interventions. By identifying patterns and factors affecting these changes we can develop more effective rehabilitation protocols to improve our patient’s quality of life and function.